# High-content image-based screening reveals vaccinia virus membrane proteins as potential regulators of LC3 lipidation

**DOI:** 10.1128/spectrum.01221-26

**Published:** 2026-09-23

**Authors:** Melanie Krause, Artur Yakimovich, Noemi Vágó, Ingo Drexler, Jason Mercer

**Affiliations:** 1 MRC Laboratory for Molecular Cell Biology, University College London 4919, London, United Kingdom; 2 Center for Advanced Systems Understanding (CASUS) 682670, Görlitz, Germany; 3 Helmholtz-Zentrum Dresden-Rossendorf e. V. (HZDR) 28414 , Dresden, Germany; 4 Institute of Computer Science, University of Wrocław 495570, Wrocław, Poland; 5 Cluster of Excellence Physics of Life, TU Dresden, Dresden, Germany; 6 Institute of Computer Science, University of Wrocław, Düsseldorf, Germany; 7 Institute of Microbiology and Infection, School of Biosciences, University of Birmingham, Birmingham, United Kingdom; Regional Centre for Biotechnology, Faridabad, Haryana, India

**Keywords:** autophagy, poxvirus, infection, immunity, LC3, high-content screening, fluorescent image analysis

## Abstract

Autophagy is a catabolic process used for the degradation of organelles and proteins, and it also functions as a cellular defense mechanism, known as xenophagy, during infection. Vaccinia virus (VACV), the prototypic poxvirus, replicates exclusively in the cytoplasm of host cells. It is known that VACV infection causes LC3 lipidation while preventing autophagosome formation, yet the viral protein(s) responsible have not been identified. Using infection time-course Western blotting and a phospholipase D band-shift assay, we show that VACV drives genuine LC3-II formation, rather than a block in pro-LC3 cleavage, and that this depends on early viral gene expression. To identify the gene(s) involved, we generated a dsRed-LC3-I-GFP reporter cell line and developed an image-based screening approach based on single-cell LC3 granularity, which we applied to 80 early VACV genes. The screen revealed that VACV-induced lipidation is regulated in both directions. Follow-up validation identified H3 as a negative regulator and the membrane proteins A14, L5, and G5 as positive contributors to LC3 lipidation. These findings provide the first systematic map of VACV genes affecting LC3 lipidation and suggest that the interplay between VACV and autophagy is more directed than previously thought.

**IMPORTANCE:**

Poxviruses, including vaccinia virus and the closely related monkeypox virus, remain a significant public health concern and are increasingly exploited as vaccine and cancer immunotherapy vectors. Understanding how these viruses manipulate host defenses is therefore important for both antiviral and biotechnology applications. Autophagy is a key cellular defense pathway, and vaccinia virus is known to trigger lipidation of the autophagy protein LC3 while blocking productive autophagy, although the viral genes driving this remained unknown. Using an image-based screening strategy, we identify several early vaccinia virus genes that increase or decrease LC3 lipidation, showing that the virus actively regulates this process in both directions. These findings reveal a more targeted interaction between vaccinia virus and host autophagy than previously recognized and provide a broadly applicable screening approach for studying virus-autophagy interactions.

## INTRODUCTION

Vaccinia virus (VACV) is a double-stranded DNA virus that replicates exclusively in the cytoplasm of its host cell. It was used as a vaccine agent to successfully eradicate smallpox. However, in 2022, a closely related virus - monkeypox (mpox) - caused a global outbreak with 91,000 confirmed human cases in 115 countries (1, 2). With a mortality rate of up to 15%, understanding the biology of mpox and other clinically relevant poxviruses continues to be crucial for treating human disease (3). Additionally, VACV and several VACV-derived poxvirus strains are used as vehicles for immunotherapy in cancer research and vaccine delivery. This necessitates continued research to further understand the interactions between VACV, its host, and cellular immune responses.

An important part of the cellular immune response to pathogens residing in the cytoplasm, such as VACV, is autophagy. Macroautophagy (hereafter referred to as autophagy) provides protective immunity by capturing pathogens in autophagosomal compartments, which later fuse with lysosomes to mediate the degradation of pathogens and other unwanted cytoplasmic content (4). Beyond its role in immunity, autophagy is a fundamental homeostatic process that maintains normal cellular function through the turnover of proteins and organelles, the recycling of nutrients, and the regulation of cell survival under stress (5). Accordingly, its dysregulation is implicated in a wide range of pathological conditions, including cancer, neurodegeneration, and infectious and metabolic diseases (6).

ATG8 (in yeast) and ATG8-family proteins (in mammals) are crucial for functional autophagy. In mammals, at least seven ATG8 orthologs exist. The most studied ATG8 ortholog is LC3b (hereafter LC3), which is critical for the execution of autophagy and often used as a marker for autophagic activity and flux (7).

Two essential ubiquitin-like conjugation systems drive autophagy to covalently conjugate ATG8-family proteins to the phagophore membrane. The first complex crucial for this process is the ATG12-ATG5-ATG16L1 complex. The carboxy-terminal glycine of the small ubiquitin-like protein ATG12 is activated by transient linkage first to the E1-like enzyme ATG7 and then to the E2-like enzyme ATG10, before becoming covalently attached to ATG5 (8, 9). This ATG12-ATG5 complex non-covalently associates with the protein ATG16L, forming the ATG12-ATG5-ATG16L1 complex that participates in the second ubiquitin-like conjugation reaction (10). The second conjugation step results in the covalent addition of the lipid phosphatidylethanolamine (PE) to LC3. This process, known as ATG8/LC3 lipidation, is mediated by the E1-like ATG7 and E2-like ATG3 enzymes. The carboxy-terminal amino acids of LC3 are cleaved by the cysteine protease ATG4, presenting a conserved glycine residue. Cleaved LC3 can then be transiently linked to ATG7, then to ATG3, and finally to PE. The role of an E3-like ligase is filled by the ATG12-ATG5-ATG16 complex (11).

Although a complete lack of autophagosome formation is found in VACV-infected cells, previous research showed that VACV infection leads to LC3 lipidation independent of the presence of ATG5 or ATG7 (12). While ATG5- and ATG7-independent autophagy exists and has been shown to mediate autophagic degradation of *Shigella* in intestinal cells (13), it does not typically involve LC3 lipidation (14, 15). Moloughney et al. also observed that VACV infection causes ATG12-ATG3 conjugation in the absence of ATG5 and ATG7 and that these ATG12-ATG3 conjugates cluster with viral DNA (12). They proposed a model whereby ATG12-ATG3 complexes drive aberrant LC3 lipidation due to spatial dislocation from the phagophore, resulting in a failure of autophagosome formation. The viral protein causing this complex formation has not been identified.

In this work, we set out to identify candidate viral genes involved in increased LC3 lipidation employing an image-based siRNA screen of 80 early VACV genes. To quantify LC3^+^ autophagosomes, we used a readout based on single-cell granularity (16–18). The core methodology involves a pipeline starting with cell nuclei and cytoplasm segmentation (16), followed by single-cell granularity analysis (17, 18) employing dsRed-LC3 fluorescence. We identified five VACV genes that upregulate and nine genes that downregulate LC3 lipidation. Conformational siRNA depletion coupled with immunoblot analysis for LC3 lipidation showed that H3, A14, L5, and E8 influence LC3 lipidation during infection. This study delivers the first systematic analysis of VACV genes intersecting with LC3 lipidation and provides the starting point for detailed follow-up analysis of VACV-mediated modulation of LC3 lipidation.

## RESULTS

### VACV infection leads to the accumulation of LC3^+^ puncta

**Fig 1:**
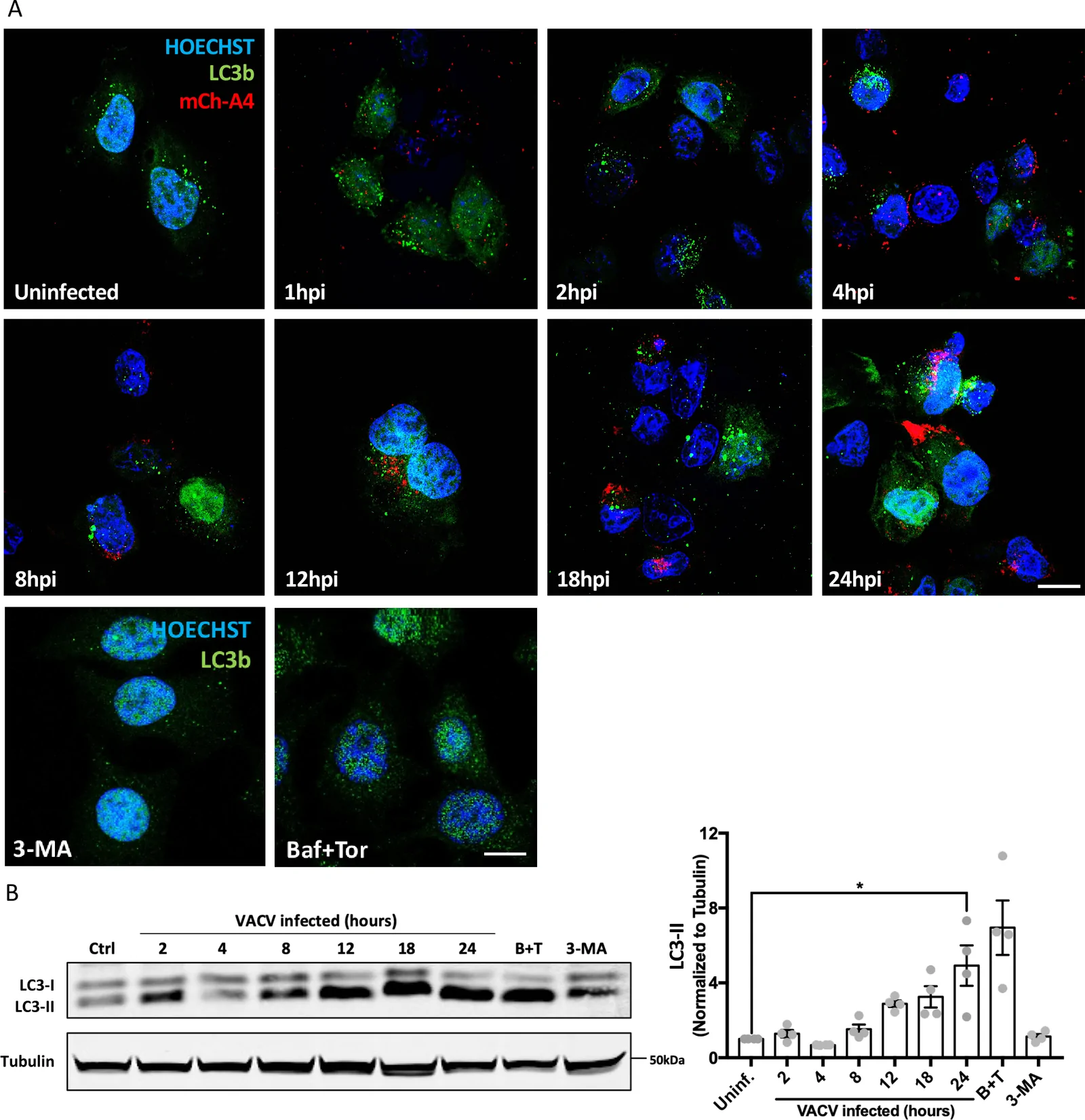
VACV infection causes an increase in LC3b puncta formation and in LC3-II. (**A**) HeLa cells were infected with VACV for the times indicated and stained for LC3b or treated with 3-methyladenine (3-MA), BafilomycinA1, and Torin1 at standard concentrations for 4 h. Representative images of three biological replicates are shown. Scale bars represent 10 µM. (**B**) HeLa cells were infected with VACV or incubated with Bafilomycin A1 and Torin1 (B+T) or 3-MA for 8 h. Right: quantification of four biological repeats of the experiment shown. Statistical analysis: unpaired *t*-test with *P < 0.05. Error bars represent SEM.

To confirm the previous finding that VACV induces LC3 lipidation, we monitored a 24 h infection time course. VACV infection led to the accumulation of LC3^+^ puncta, indicative of an enrichment of autophagosomes. We observed a slight increase in LC3^+^ puncta during VACV infection. While at late time points post-infection viral DNA replication sites appeared to show some minor overlap with cytoplasmic LC3, we did not observe direct co-localization between LC3 and newly formed virions. Interestingly, accumulation of LC3^+^ puncta occurred very early in infection and was already visible at 1 hpi (Fig. 1A).

### VACV infection causes increased LC3 lipidation

Moloughney et al. described an increase in LC3-II during VACV infection at 24 hpi, assuming an increase in lipidated LC3 (12). To validate these findings and determine the flux of LC3 lipidation, we performed an infection kinetics assay with subsequent Western blot analysis. Bafilomycin A1- and Torin1-treated samples were used as positive controls and 3-MA-treated samples as a negative control for LC3 lipidation. Consistent with Moloughney et al*.,* LC3-II was increased in VACV-infected cells. Quantification showed the increase to occur gradually over time, with fivefold more 14 kDa LC3 being present in cells 24 hpi compared to uninfected control samples (Fig. 1B).

However, when visualizing LC3 protein using Western blotting, two bands at 16 kDa and 14 kDa can be observed. These bands are indicative of three different states of LC3: pro-LC3, LC3-I, and LC3-II. To be lipidated, pro-LC3 (14 kDa) is converted to LC3-I (16 kDa) through cleavage of carboxy-terminal amino acids by ATG4. PE is then linked to the 16 kDa LC3-I, converting it into LC3-II (14 kDa). As pro-LC3 and LC3-II both migrate at 14 kDa by Western blot, an increase in 14 kDa LC3 can be attributed to either an increase in LC3-II formation or accumulation of pro-LC3 caused by a block in cleavage.

To differentiate between pro-LC3 and LC3-II during VACV infection, we utilized a phospholipase D (PLD) assay (19). This assay relies on the ability of PLD to cleave LC3-II, but not pro-LC3. Incubating cell lysate with PLD shifts LC3-II from 14 kDa to 16 kDa, while pro-LC3 remains unaffected. PLD-cleaved LC3 cannot be lipidated again due to the different cleavage sites of ATG4b and PLD; thus, a shift of LC3 from 14 kDa to 16 kDa in the presence of PLD indicates that the increased 14 kDa LC3 is due to LC3-II formation.

**Fig 2:**
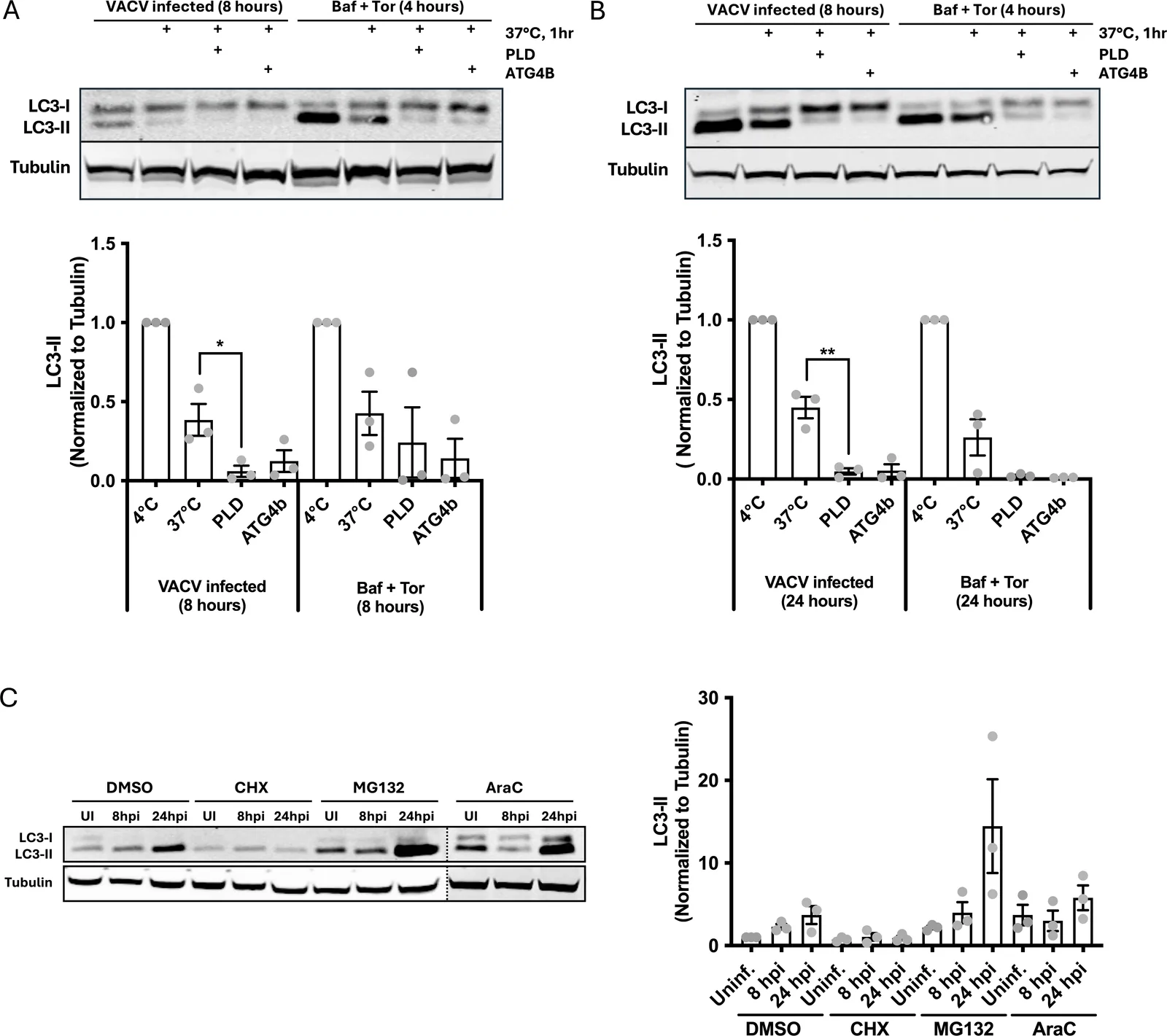
VACV infection causes LC3 lipidation, not pro-cleavage block, through expression of an early viral gene. (**A**) PLD assay carried out for VACV WR WT infection at 8 hpi with bafilomycin A1 and Torin1 ctrl with quantification of *n* = 3 biological replicates below. (**B**) PLD assay carried out for VACV WR WT infection at 24 hpi with Bafilomycin A1 and Torin1 ctrl with quantification of *n* = 3 biological replicates below. (**C**) HeLa cells were infected with VACV WR WT at MOI 30 for the times indicated in the presence of 50 μM CHX, 25 μM MG132, 10 μM AraC, or DMSO, with quantification of *n* = 3 biological replicates on the right. Statistical analysis: unpaired *t*-test with *P < 0.05 and **P < 0.01. Error bars represent SEM.

The PLD cleavage assay was performed at two different time points to differentiate between a pro-cleavage block and LC3-II formation during both early and late stages of VACV infection. The earliest time point with an increase in lower-band LC3 sufficient for visualization was at 8 hpi, while 24 hpi was chosen as the late time point representing the peak of VACV-mediated LC3 modification in WB-based time-course experiments. While incubation at 37°C led to some degradation of LC3, enough remained to be used as a functional readout. ATG4b, which cleaves both LC3-II and pro-LC3, was used as a positive control. An additional control used in this assay was uninfected lysate treated with Bafilomycin A1 and Torin1, which causes LC3-II, resulting in a band shift to LC3-I upon PLD incubation. Importantly, PLD treatment of either VACV-infected or Bafilomycin A1 + Torin1-treated samples displayed an LC3 band shift, with the lower band shifting upward, indicating that the lower LC3 band mainly consists of LC3-II. This was documented for both time points at 8 and 24 hpi during VACV infection (Fig. 2A and B). This shows that VACV causes LC3 lipidation throughout the course of infection rather than blocking pro-LC3 cleavage.

### One or more early VACV genes facilitate LC3 lipidation

To narrow down the search for the viral gene(s) initiating LC3-II formation, we looked at VACV-induced LC3 lipidation in the presence of three drugs known to interfere with the VACV life cycle. Cells were infected with WT VACV (MOI 3) in the presence or absence of cycloheximide (CHX; 50 μM), MG132 (25 μM), or cytosine arabinoside (AraC; 10 μM), all of which are used in VACV research for temporal inhibition of the VACV life cycle at distinct stages. CHX prevents early viral protein synthesis without impacting virus entry and core release. MG132 prevents viral uncoating through proteasome inhibition while allowing for viral early gene expression (20–22). AraC allows for uncoating and early gene expression, but blocks intermediate and late gene expression by interfering with viral DNA synthesis (23).

HeLa cells were infected with VACV WR WT, treated with CHX, MG132, or AraC, and LC3-II formation was analyzed by WB. MG132 and AraC did not prevent LC3 lipidation during infection. CHX, however, completely abrogated LC3-II formation, suggesting an early viral gene product was responsible (Fig. 2C).

### Assessing LC3-II lipidation during VACV infection using a dsRed-LC3-GFP marker cell line

While flow cytometry provides information regarding intensity and cell granularity, an image-based readout can provide additional single-cell information, including the number, size, and shape of LC3 puncta, and thus, is a preferable readout. To visualize the formation of LC3^+^ autophagosomes, we constructed a modified dsRed-LC3-I-GFP HeLa cell line expressing fluorescent LC3 generated using a pQCXI-Puro-dsRed-LC3-GFP construct (24), which is a modified version of another LC3 construct (25).

**Fig 3:**
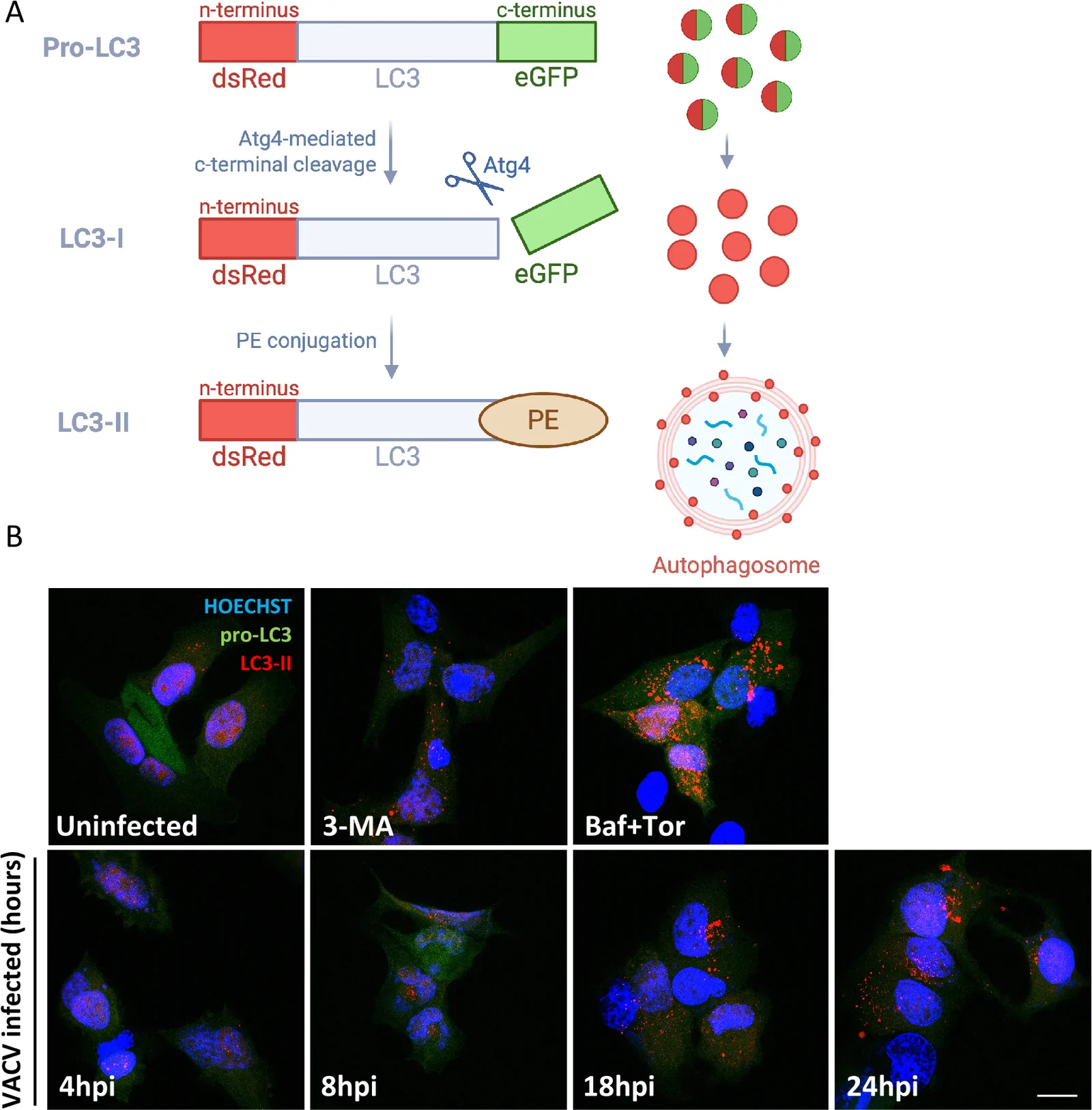
A cell line expressing a dsRed-LC3-I-GFP construct is used as a readout for LC3 lipidation. (**A**) Schematic representation of dsRed-LC3-I-GFP. (**B**) Representative confocal images of the cell line expressing the dsRed-LC3-I-GFP construct shown in panel **A**. HeLa cells were treated with Bafilomycin A1 and Torin1 or 3-MA at standard concentrations for 4 h or infected with VACV. Scale bar indicates 15 μM.

Here, LC3 bears an N-terminal dsRed tag and a C-terminal eGFP tag. As mentioned, during LC3 processing the c-terminus is modified by ATG4 cleaving the protein at the location indicated in Fig. 3A leading to removal of the GFP tag. This cleavage process is necessary to attach PE to LC3 and to conjugate it to the autophagosome. Thus, LC3-II appears only as red, making it screenable in both immunofluorescence (IF) and flow cytometry-based readout systems. Similar constructs have been published and used for various applications in autophagy research, such as to screen for autophagy-inducing drugs with LC3 as a readout for autophagic flux (26, 27).

Cells were either treated with autophagy-modulating drugs or infected with VACV. While only a few red autophagosomal puncta were present in untreated and uninfected cells, treatment with a combination of Bafilomycin A1 + Torin1 drastically increased the number of LC3^+^ autophagosomes. Additionally, we observed a steady increase in LC3^+^ puncta during a time course of VACV infection, peaking at 24 hpi (Fig. 3B). These results confirmed that VACV infection induces LC3 lipidation and indicated that an image-based screening using this cell line as a readout for LC3-II formation is feasible. Interestingly, LC3 appears to show partial nuclear accumulation following Bafilomycin A1 + Torin1 and 3-MA treatment. Huang et al. showed that the nuclear pool of LC3 is the primary source for autophagosome-conjugated LC3 and that this nuclear-to-cytoplasmic redistribution feeds autophagosome formation (28), suggesting that by blocking nucleation/early flux, 3-MA prevents LC3 from leaving the nucleus to form puncta. However, the reporter may also cause artifacts, as overexpressed LC3 can form aggregates and puncta independently of autophagy (29). Given the diffuse nucleocytoplasmic distribution of soluble LC3 and the aggregation affinity of dsRed, apparent nuclear accumulation should be interpreted with caution.

To screen for the early viral gene(s) impacting LC3-II formation during VACV infection in the dsRed-LC3-I-GFP HeLa cell line mentioned above, we utilized a VACV early gene siRNA library (20), which targets 80 VACV early genes with three individual siRNAs per gene.

### Development of a screening system measuring LC3-II granularity

The VACV early gene-LC3 lipidation screen was carried out by transfecting HeLa dsRed-LC3-I-GFP cells with siRNAs directed against 80 early VACV genes (20, 30). Prior to infection, cells were reverse-transfected with siRNAs in 96-well plates and incubated for 16 h, which is sufficient for early VACV gene depletion (20, 31, 32). Images were obtained using the Opera Phenix High-Content Screening System. Next, image segmentation of individual cells was performed using a custom preprocessing script and a CellProfiler pipeline (16).

**Fig 4:**
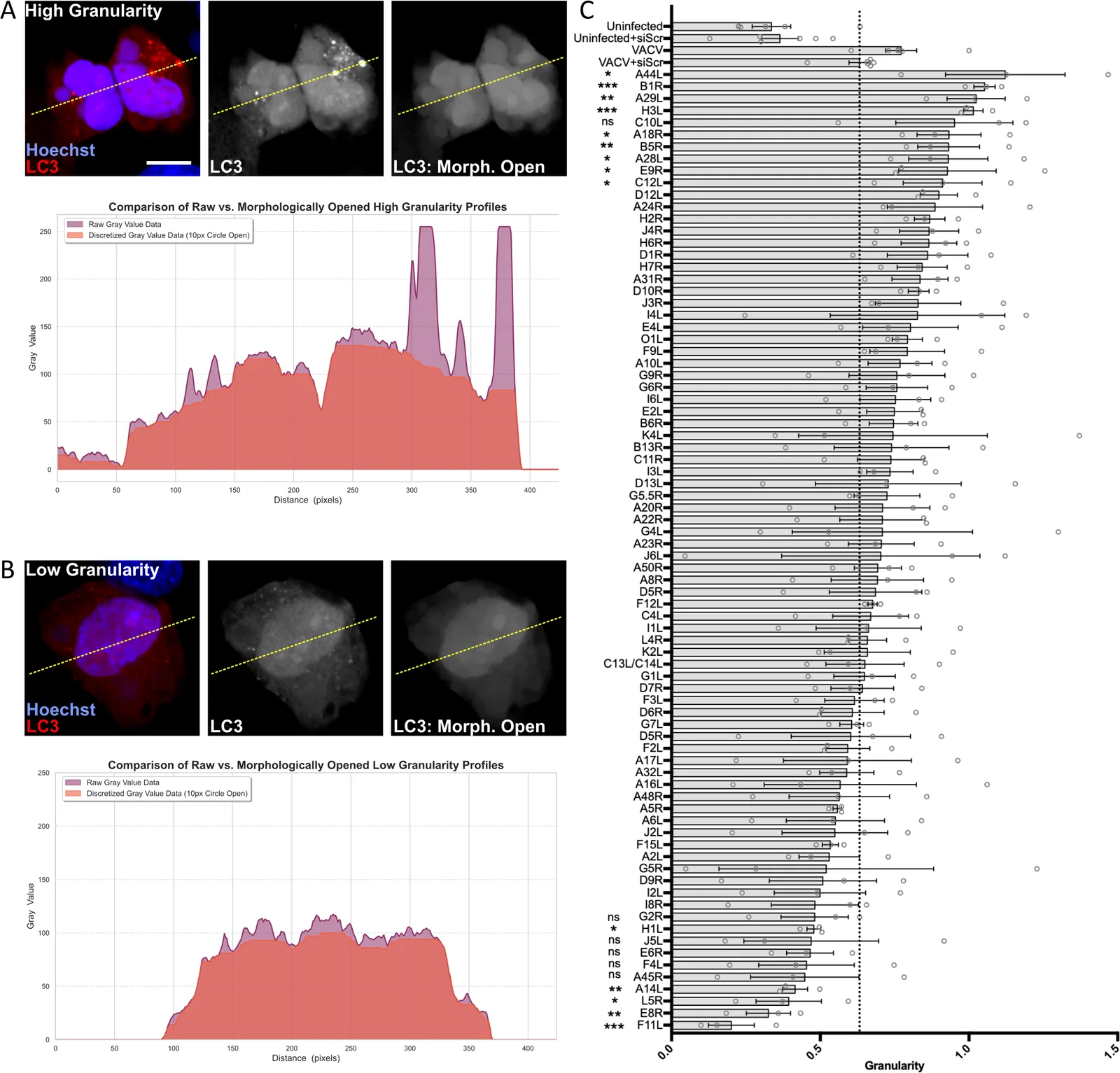
An early VACV gene siRNA screen identifies several candidates for VACV-mediated increase and decrease of LC3 puncta formation. (**A and B**) Principal approach to granularity measurement. (**A**) Top: an example of a high-granularity cell. (**B**) Top: an example of a low-granularity cell. Left-to-right channel merge, LC3 channel, and morphologically processed LC3 micrograph using ray morphology open with a 10 px circle as a structuring element. The yellow dashed line corresponds to the position where the linear profile was measured. (**A, B**, bottom) Line profile intensity measurement of unprocessed (raw) and processed micrographs of high and low granularity, highlighting the principal approach of granularity quantification. (**C**) Cells were treated with siRNA for 16 h and infected with WR WT at MOI 10. Red puncta of lipidated LC3 were detected and used for granularity readout. The figure shows pooled results for all three siRNAs per gene, with each dot representing an *n* = 3 for one specific siRNA. Error bars indicate SEM. The scale bar indicates 10 µm. Statistical analysis: Unpaired *t*-test with *P ≤ 0.05, **P ≤ 0.01, and ***P ≤ 0.001.

Once individual cells were segmented, several single-cell readouts were tested, such as the average intensity of the dsRed signal, total intensity of the dsRed signal, median intensity of the dsRed signal, and granularity spectrum (see Materials and Methods). The latter was chosen as the readout as it most accurately reflects the presence or absence of autophagosomes within the cell, with the granules indicating LC3^+^ autophagosomes (Fig. 4A and B). In this readout, the better the selected structuring element size fits the signal, the higher the score. This makes it possible to detect the occurrence of larger granules in the cytoplasm of the cell upon adjustment of the structuring element size hyperparameter. Thus, this is a single-cell-level readout of granule formation. High-granularity structures correspond to the highest peak difference upon morphological opening with a structuring element of a fitting radius (Fig. 4A and B).

### Identification of potential viral regulators of LC3 lipidation

For controls in this screen, we used untreated cells which were either infected (labeled as VACV) or uninfected, as well as cells treated with siScramble, also either infected or uninfected. While siScramble had no effect on LC3 granularity, VACV infection nearly doubled the granularity score both in the presence and absence of siScramble compared to uninfected cells (Fig. 4C). The granularity score for VACV treated with siScramble was slightly lower compared to untreated VACV-infected cells. While we expected to find that depletion of some VACV gene products would result in reduced LC3 puncta, we also observed that depletion of some VACV early gene products led to an increased granularity score. This suggests that VACV-mediated LC3 lipidation is regulated in both directions.

Applying VACV untreated as a threshold resulted in 53 screen hits with an increased and 27 screen hits with a decreased LC3 granularity score. Applying VACV siScramble KD as a control (indicated by the dotted line in Fig. 4C) results in 25 screen hits with increased LC3 granularity and 55 with a decreased LC3 granularity score.

Statistical analysis was carried out to decide which genes to select for follow-up experiments. For the genes whose gene product depletion resulted in a granularity increase, we selected the four highest-scoring genes A44L, B1L, A29L, and H3L. Additionally, A28L was selected based on the performance of siRNA 1. For hits showing decreased granularity, we selected the four lowest scoring genes A14L, E8R, L5R, and F11L. Additionally, G5L was also selected based on the performance of siRNA 1.

### VACV H3, G5, L5, and A14 may mediate VACV-induced LC3 lipidation

**Fig 5:**
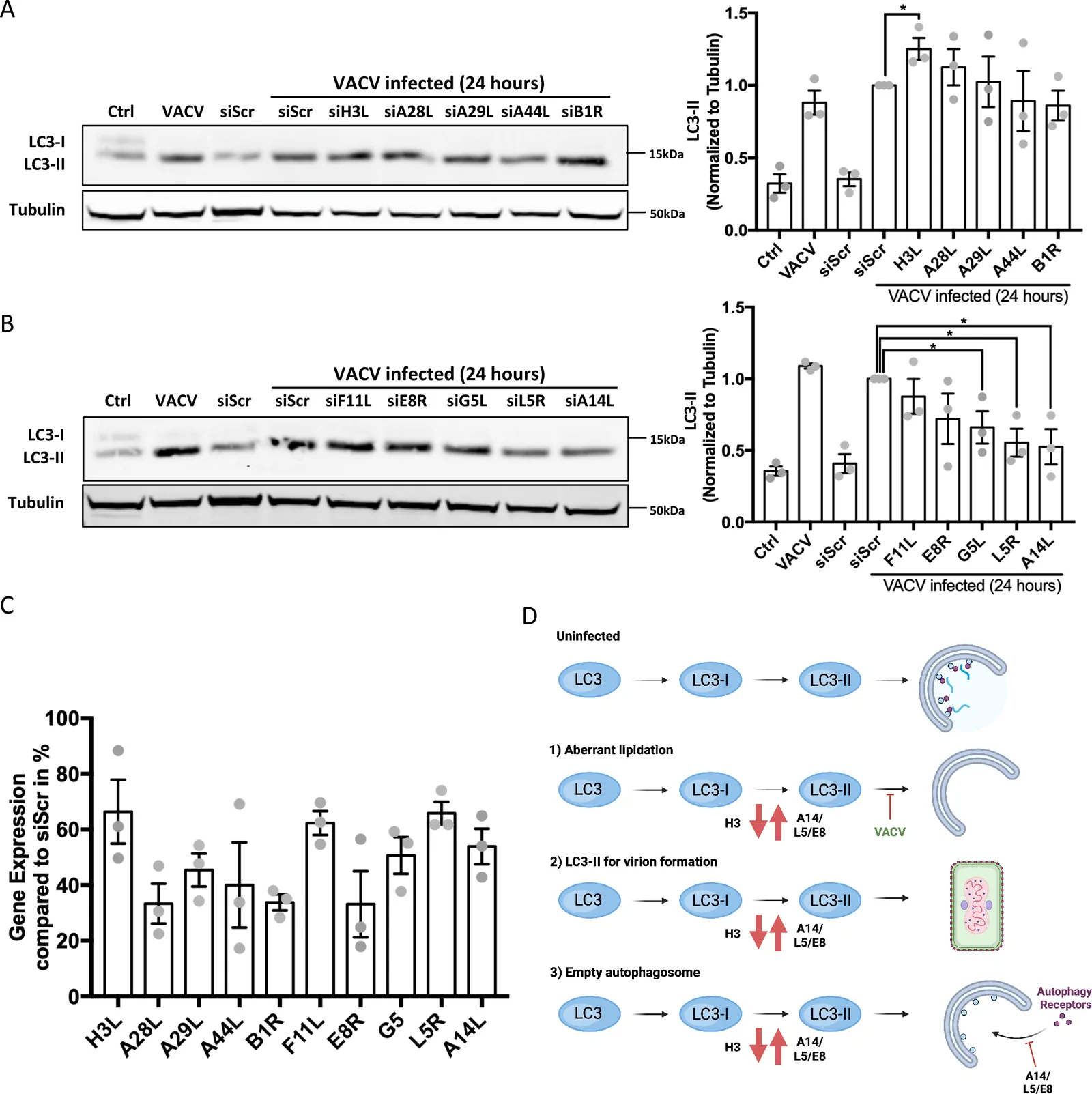
Hit validation of LC3 screen. (**A**) Validation of hits for LC3 lipidation decrease, sample Western blot (left) and quantification of three Western blots (right). (**B**) Validation of hits for LC3 lipidation increase, sample Western blot (left) and quantification of three Western blots (right). (**C**) Quantitative real-time PCR (qRT-PCR) validation of KD efficiency, *n* = 3 independent repeats in comparison to gene expression in siScr samples. Statistical analysis of *n* = 3 repeats for panels **A and B**: unpaired *t*-test with *P < 0.05. All controls are significant with at least ***P < 0.0001. The absence of significance indication in target genes means non-significant. Error bars represent SEM. (**D**) Three different models of LC3 lipidation: (1) A viral protein causes ATG12–ATG3 conjugation. The ATG12–ATG3 complex results in aberrant LC3 lipidation dislocated from the phagophore, leading to failure in autophagosome formation. (2) Lipidated LC3 contributes to the formation of new virions. (3) LC3 lipidation is a side effect of autophagy activation during infection due to depletion of autophagy receptor proteins.

To validate the screening results and to confirm that the LC3 puncta seen in the screen correspond to the LC3 lipidation results, the various candidate gene products were depleted using their respective strongest-performing siRNA. WB-based LC3 lipidation assays were carried out. Since cells were plated at reduced confluency for siKD compared to other WB samples, the MOI was increased to six to maintain the LC3 lipidation phenotype. The WB results indicated that H3 is the strongest candidate for VACV-mediated negative regulation of LC3 lipidation. Depletion of H3 resulted in an even stronger lipidation phenotype than siScramble, with an increase of 25% (Fig. 5A).

In contrast, LC3 lipidation was significantly reduced upon siRNA-mediated depletion of G5, L5, and A14 protein synthesis. A14 KD resulted in a reduction of LC3 lipidation by nearly 50% when compared to VACV-infected siScramble samples. Interestingly, the uninfected siScramble control exhibited 60% less LC3 lipidation than VACV siScramble. Reduction of A14, therefore, nearly abrogated the LC3 lipidation phenotype of VACV compared to uninfected siScramble-treated cells. Other gene products exposed to siRNA-mediated decay resulted in a significant reduction of LC3 lipidation, such as L5 (reduction of lipidation by 45% compared to VACV siScramble) and G5 (reduction of lipidation by 33% compared to VACV siScramble) (Fig. 5B).

To ensure that the siRNA used in these assays could efficiently deplete the respective mRNA and diminish target protein synthesis, we used qRT-PCR (Table 1) to validate the KD efficiency of the siRNAs (Fig. 5C). All siRNAs showed some level of depletion of their respective target mRNAs to varying degrees. The average reduction of mRNA transcripts for H3L, F11L, and L5R was 30%, while siRNAs for A28L, B1R, and E8R achieved a KD efficiency of 70%. Thus, H3 depletion might have a stronger impact on LC3 lipidation than suggested by Fig. 5A, but limited siKD efficiency prevented full manifestation of the phenotype in WB analysis. Likewise, the reduction of LC3 lipidation in siL5R- and siA14L-treated samples might be more prominent if the KD efficiency was higher.

**TABLE 1:** List of qRT-PCR primers

Primer name	Sequence (5′−3′)
E8R FWD Set 2	AAG GCT GGC GAC ATT TAG AG
E8R REV Set 2	GTG TGG GCG ATT ACC TTT CT
G5R FWD Set 3	GTA CAG ACC AGG TGG CTT AAA
G5R REV Set 3	CGG TAC ATT CAG CCT CGT T
L4R FWD Set 4	GAA CCT TAG CCG TTC AGA TGT A
L4R REV Set 4	TCT AAG GAG CAG GGA ACA ATT T
H3L FWD Set 4	GGA TAC TCA CGT GAA GGT CTT G
H3L REV Set 4	GAT TAT TCC AGT TGG GCT TGT TG
A28L FWD Set 5	ATG AGG CAA GGC GAG AAG
A28L REV Set 5	CAG TGG TAC GAC CTG ATA GAT G
A29L FWD Set 5	TCT ACC AGG AAG AAC TAG CAA ATC
A29L REV Set 5	CAA ACA GCA CAA CCA GTG TC
A44L FWD Set 5	TCG GGC AGA GGA AGT TCT AT
A44L REV Set 5	GCA CGA GGA TTT CTC AGA AGG
B1R FWD Set 3	GTT TCC GGA TCC TCT GAG TTT
B1R REV Set 3	GGA GAG TGT TGG AAT GAG TGT
F11L FWD Set 1	GAG GCT TCA TGC TGG GAT AC
F11L REV Set 1	GTA CCA CAG TCC TTG GAT TAC C
A14L FWD Set 2	GTT AGA CCA CAG CAG AGA AGT C
A14L REV Set 2	GGC ACG AGG GCT TTA ATA CT
GAPDH fw (RT-PCR, Ctrl)	AAG GTC GGA GTC AAC GGA TTT GGT
GAPDH rv (RT-PCR, Ctrl)	ACA AAG TGG TCG TTG AGG GCA ATG

## DISCUSSION

Autophagy is of central importance in host-virus interactions. Many viruses actively suppress the pathway to evade xenophagy. The herpes simplex virus 1 neurovirulence factor ICP34.5, for example, binds Beclin 1 to block autophagosome formation and promote neurovirulence (33). Analogous Beclin 1-binding Bcl-2 homologs are encoded by γ-herpesviruses such as KSHV and murine γ-herpesvirus 68 (34, 35). Other viruses co-opt the pathway to support replication: Poliovirus subverts the autophagosome-formation machinery to generate membranes that serve as platforms for viral RNA replication (36). Hepatitis C virus and influenza A virus induce autophagosome accumulation while blocking their maturation and uncoupling LC3 lipidation from productive degradation (37, 38).

The latter scenario is relevant to our findings, as the accumulation of LC3-II we observe during VACV infection also appears dissociated from canonical autophagic flux. Notably, VACV is unusual among these examples, as it drives LC3 lipidation without supporting autophagosome formation (12), suggesting a mechanism distinct from the membrane-scaffolding strategies described above and consistent with our proposal that VACV engages the LC3 conjugation machinery in a more directed manner than previously recognized.

Notably, both in Western blot and microscopy analysis, we observe a reduction in LC3-II at 4 hpi (Fig. 1). While not statistically significant, it might indicate that the early LC3 lipidation increase at 2 hpi could be a transient host response to virus entry and sensing rather than to viral gene products. Here, VACV enters by macropinocytosis, causing membrane and osmotic perturbation. The cell might initially attempt to mount xenophagy, which could produce a short-lived LC3-II spike. This idea is supported by ring formation of p62/SQSTM1 around incoming VACV (39). Then, as the virus establishes infection and begins remodeling host signaling, that host-driven component subsides at 4 hpi, after which the virus-driven, early-gene-dependent aberrant lipidation takes over and accumulates progressively to 24 hpi. In this case, the first phase would be host-initiated and transient, the second virus-initiated and sustained.

Another option might be if lysosomal degradation is still fully functional at 2–4 hpi; the early LC3-II made in response to entry could be turned over, whereas later in infection, a block on autophagosome maturation/fusion or depletion of degradative capacity would cause net LC3-II to accumulate regardless of the rate of formation. Our work, combined with the A52–SNAP29 autolysosome block (40) and VACV-induced depletion of autophagy receptors (39), might predict a shift toward accumulation at later times.

In this screen, siRNA-mediated depletion of several early VACV candidate genes was associated with altered LC3 lipidation. We emphasize, however, that the knockdown efficiency achieved for some targets was modest (~30% for H3L and L5R; Fig. 5C), so the resulting assignments should be regarded as provisional and as candidates for mechanistic follow-up rather than as established effectors. Because partial depletion is expected to understate rather than overstate the underlying contribution, we interpret the significant phenotypes obtained under these conditions as a conservative readout; nonetheless, further analysis with more complete depletion or complementary loss-of-function approaches will be necessary to confirm these candidates and to define their interaction with LC3 in detail. While the LC3 signal is specific to this screen, our granularity-based approach could, in principle, be used to interrogate related phenotypes such as endosomal structures or multivesicular bodies, and may therefore be applicable to other high-content image-based screens.

Because the increase reflects genuine LC3-II rather than a block in pro-LC3 processing, and because the resulting puncta could correspond either to *bona fide* autophagosomes or to aggregates of lipidated LC3 (12), we propose three non-exclusive models for the role of VACV-induced lipidation (Fig. 5D). First, the lipidation may be aberrant, as suggested by Moloughney et al. This could represent an autophagy-evasion strategy: As cargo is recruited into the forming autophagosome via autophagy receptors which bind to LC3, aberrant LC3 lipidation would impede this process, leaving forming autophagosomes “empty” and non-functional. Second, lipidated LC3 may contribute to the formation of new virions. It was originally thought that VACV membranes may utilize the autophagy machinery for membrane formation (41). While Beclin 1, ATG5, and ATG7 have been excluded as requirements for VACV membrane formation (41), a specific role for lipidated LC3 has not been tested. Third, LC3 lipidation may be a by-product of autophagy activation upon sensing of incoming virions, with the VACV-induced depletion of autophagy receptors (39) leaving an excess of autophagosomal membranes.

The identity of the candidate regulators may shed further light on this. siRNA targeting H3 is the only treatment which significantly increased lipidation. Exogenous heparin, a structural analog of heparan sulfate, has been reported to suppress hyperglycemia-induced autophagy in cultured mesangial cells and in diabetic rats, where chronic heparin treatment reduced LC3-associated autophagic responses (42). Although the mechanisms are likely to differ, these observations raise the possibility that interactions between H3 and heparan could influence signaling pathways that regulate LC3 lipidation during VACV infection.

Among the genes whose KD reduced lipidation, A14, L5, and G5 were confirmed by Western blot. A14 is essential for viral membrane biogenesis and a major component of the mature virion membrane. Together with A17, it forms viral membrane crescents which progress into the immature virion membrane. Both also form the disulfide-stabilized lattice anchoring other virion proteins (43–45). L5 is a membrane component of the entry-fusion complex required for cytoplasmic entry (46, 47), and G5 is thought to be a putative endonuclease required for double-strand break repair, homologous recombination, and production of full-length viral genomic DNA (48–50). While E8 siKD did not lead to a significant decrease in VACV-mediated LC3 lipidation by WB, it remains of interest as an ER-localized membrane protein, given that ER stress both induces autophagy and supplies LC3 precursors (51, 52). That three of the five selected candidates are membrane proteins is striking and hints at a link between LC3 lipidation and viral membrane formation.

This is consistent with several features of the VACV life cycle. Early replication sites become enwrapped by ER membranes, essential for efficient DNA synthesis (53), placing replication near sites of autophagosomal origin. We have previously shown that VACV exploits ESCRT-mediated multivesicular bodies during virion wrapping (54); aberrant autophagosomal structures could provide an additional membrane source for this process. Mechanistically, LC3 lipidation during VACV infection proceeds even in ATG5- and ATG7-deficient cells (12). Thus, Moloughney et al. proposed that a viral protein substitutes for the E3-like ATG12-ATG5-ATG16L activity that normally conjugates LC3 to PE. A recent publication further suggests that VACV A52 blocks autolysosome formation by binding SNAP29 (40), adding another layer of viral control over the pathway.

Taken together with the existing literature, our findings indicate a closer and more directed interplay between VACV and the host autophagy machinery than previously assumed. Deciphering the involvement of these viral proteins in abrogating autophagy will be an important avenue for future research.

## MATERIALS AND METHODS

### Cell culture and viruses

HeLa cells (WT as well as a HeLa-based fluorescent cell line) were maintained in Dulbecco’s Modified Eagle Medium (DMEM, Life Technologies) supplemented with 10% fetal bovine serum (FBS, Life Technologies), 1 mM sodium pyruvate (Thermo Fisher Scientific), 100 μM non-essential amino acids, 2 mM L-alanyl-L-glutamine dipeptide (GlutaMAX, ThermoFisher), and 1% Penicillin-Streptomycin (Life Technologies) at 37°C and 5% CO_2_. Cell lines were maintained in either T75 or T175 flasks and passaged two to three times per week using phosphate-buffered saline (PBS) and trypsin/EDTA (2.5 g Trypsin/L, 0.2 g EDTA/L). The following viruses were used: wild-type Western Reserve strain (VACV WR WT) and VACV WR encoding an endogenous C-terminal mCherry-tagged A4 core protein (WR mCherry-A4) (22, 55).

### Reagents and antibodies

AraC and CHX were obtained from Sigma-Aldrich, while MG132 was purchased from Merck Millipore. The drugs were used at 10 μM, 50 μM, and 25 μM, respectively. CHX inhibits protein synthesis and thereby blocks early viral protein production; MG132 inhibits the proteasome and prevents viral core uncoating while still permitting early gene expression (20–22); and AraC inhibits viral DNA synthesis, allowing uncoating and early gene expression but blocking intermediate and late gene expression (23). Bafilomycin A1 from *Streptomyces griseus* (Sigma-Aldrich) is an inhibitor of the late stages of autophagy that blocks autophagosome-lysosome fusion and lysosomal acidification through inhibition of the vacuolar H^+^-ATPase (56). Torin1 (Merck Millipore) is an mTOR inhibitor that induces autophagy (57). In combination with Bafilomycin A1, it served as a positive control for LC3 lipidation. 3-MA (Sigma-Aldrich), an inhibitor of the class III PI3K (VPS34) that blocks the early steps of autophagosome formation (58), was used at 5 mM as a negative control for LC3 lipidation.

DMSO (Sigma-Aldrich) was used to dissolve drugs and as a negative control in all drug-related assays. Bafilomycin A1 from *Streptomyces griseus* (Sigma-Aldrich) was used at 10 nM. Torin1 (Merck Millipore) was used at 250 nM, and 3-MA (Sigma-Aldrich) was used at 5 mM. The following antibodies were used: anti-LC3b (CST #3868S) used in IF at 1:200 and in WB at 1:1,000; anti-Tubulin (CST #9701S) used in WB at 1:5,000. Hoechst 33342 trihydrochloride trihydrate (Invitrogen #H3570) was used for DNA staining in IF at 1:10,000. Secondary antibodies were purchased from Invitrogen and LI-COR.

### Imaging

For confocal imaging, cells were seeded on 13 mm glass coverslips (VWR) at 60,000 cells per coverslip the day prior to infection. Infection was carried out with WR mCh-A4 or WT virus at MOI 1 in DMEM and incubated for 1 h at 37°C. The media was removed and replaced with full medium and incubated for the desired time. Cells were fixed with 4% FA-PBS for 15 min. Cells were permeabilized with ice-cold MeOH at −20°C for 20 min, blocked with 3% bovine serum albumin (BSA, from Sigma-Aldrich) in PBS for 1 h, and stained with 30 μL primary antibody diluted in 3% BSA-PBS for at least 1 h at RT or overnight at 4°C. The coverslips were washed three times for 5 min in 3% BSA-PBS before secondary antibody and Hoechst staining for 1 h at RT in 30 μL 3% BSA-PBS and mounted on glass mounting slides. Samples were imaged using a 63× oil immersion objective (ACS APO) on a Leica TCS 2012 model SPE confocal microscope. High-content imaging was carried out as follows: prior to image analysis, cells were fixed with 4% FA. Antibody and Hoechst staining was carried out in a 40 μL volume on a shaker using dilutions as stated above. The Opera Phenix High-Content Screening System was used at 40× magnification with an air objective using 405 nm, 488 nm, 594 nm, or 647 nm lasers, and at least 15 images were taken per well. CellProfiler was used to detect individual cells based on Hoechst-stained nuclei.

### Infection time kinetics

For Western blot or fractionation samples, HeLa cells were seeded in either 60 mm or 35 mm dishes for confluency at infection. Infection was carried out in DMEM without FBS with VACV WR or any tagged virus at MOI 3. Virus was incubated with the cells for 1 h at 37°C before aspirating and replacing with full medium. Cells were either left untreated or treated with the indicated compounds from the start of infection and incubated at standard conditions. Samples were harvested at their respective time points by removing media and washing cells in PBS. Cells were harvested in lysis buffer as indicated below.

### PLD band shift assay

To determine the difference between lipidated LC3 and pro-LC3 on a Western blot, a PLD band shift assay was carried out following the previously published methodology (19). The reaction was stopped by the addition of 3× blue loading dye (CST #56036S) containing DTT and immediately boiled at 95°C for 5 min.

### Western blot analysis

Cells were washed with cold PBS and subsequently scraped into 50 μL–200 μL (depending on dish size) lysis buffer containing protease inhibitor (#5872, NEB). The sample was then left on ice for at least 20 min and subsequently spun down at 20,000 × *g* for 10 min at 4°C. The supernatant was either frozen and stored at −20°C or directly supplemented with 3× blue loading dye containing DTT. Prior to Western blot analysis, the samples were subjected to a 5 min incubation at 95°C. All protein samples were loaded into 12% Bis-Tris polyacrylamide gels and run with MES SDS buffer (both Thermo Fisher Scientific) at 60 V. Transfers were carried out onto 0.2 μm nitrocellulose membrane (Life Technologies) using the semi-dry transfer system (both Bio-Rad) at 10 V for 1 h 15 min, with transfer buffer containing methanol (NuPAGE buffer, Thermo Fisher Scientific). Primary antibodies were applied in 5% BSA TBS-T overnight at 4°C, then washed three times in TBS-T. Membranes were incubated with LI-COR secondary antibodies for 1 h at RT. Imaging of membranes was carried out with using a LI-COR Odyssey. Western blot quantifications were done using ImageJ (version 2.0.0), with Tubulin used as a loading control where applicable.

### qRT-PCR

qRT-PCR was carried out to determine the knockdown efficiency of early VACV genes that were top hits in the LC3 lipidation screen. siRNA-treated 33 mm dishes of HeLa cells were infected with WT VACV at MOI 6 for 1 h. Unbound virus was removed and replaced with full medium. Cells were harvested at 24 hpi. RNA extraction, cDNA transcription, and qRT-PCR were carried out according to a recently published protocol (59) (Table 1). Viral mRNA threshold cycle (Ct) values were calculated and expression relative to the glyceraldehyde-3-phosphate dehydrogenase (GAPDH) housekeeping gene was determined. The qRT-PCR primers used are (20, 59, 60):

### siRNA transfection

Lipofectamine RNAiMAX (Thermo Fisher Scientific) was diluted 1 in 125 in pure DMEM for 5 min at RT. siRNA dilutions and media containing Lipofectamine were then incubated together for a further 1 h at RT. The AllStars Hs Cell Death Control (Qiagen) was used as a control for transfection efficiency, and AllStars Negative (Qiagen) as an off-target, scrambled siRNA control. For Western blot analysis and confocal imaging, the siRNA-lipid complex mixture was then plated into a 30 mm dish. Three hundred thousand HeLa cells were seeded on top of the mixture in 600 μL DMEM containing FBS.

For high-content imaging of the siRNA LC3 screen, a published protocol for high-content screening of VACV early genes with an infection time of 24 h at MOI 1 (30) was used. The primers used were previously published (20).

### Computational analysis

Computational analysis of the image-based data was performed using a combination of open-source software and custom-developed code. Image analysis was performed on a desktop PC equipped with an Intel Core i7-8700K CPU at 3.7 GHz and 32 GB of RAM, as well as a GeForce 1080 Ti GPU. Image analysis for the LC3 lipidation screen was performed using a custom pipeline for the CellProfiler software (16) to detect LC3 granularity. Prior to granularity assessment, the images were processed for computational analysis: images of cell nuclei were corrected for optical shading and then convolved using a median filter (filter parameters determined by CellProfiler). Next, the sum of all channel images was produced to obtain a whole-cytoplasm signal (linear operation without hyperparameters). Subsequently, processed nuclei and total images allowed for detection of cell nuclei and cytoplasm associated with them as primary and secondary objects, respectively. Next, the granularity measurement of the LC3 signal in each cell’s cytoplasm was performed. Results of single-cell measurement were averaged per field of view.

The granularity spectrum measurement (17) used here is obtained through a morphological opening operation (18) with a disk structuring element of varying radius (ranging from 2 to 30 px) to match the cytoplasmic LC3 puncta observed in the cytoplasm of individual cells. In this approach, the best-fitting radius correlates with granule size and provides a per-cell granule size measure. The disk radius used for the readout part of the granule size spectrum, indicative of the observed phenotype, of all siRNA-treated cells was determined by comparing uninfected cells with infected cells that had not been treated with siRNA.

Based on the detected cells, LC3 signal granularity was measured for each single cell. To do so, a morphological opening operation (18) with a disk structuring element of varying radius was applied to the cytoplasmic LC3 puncta (594 nm channel) selected by the cytoplasm mask of individual cells. The disk radius used for the readout of all siRNA-treated cells was determined by comparing uninfected cells with infected cells that had not been treated with siRNA. The readout size was selected based on comparisons of LC3 granularity between infected and uninfected HeLa cells. The better the fit for the defined structuring element size, the higher the granularity score. To account for variability of the granule size, the sum of all scores per cell was used as a readout for LC3 granularity on a single-cell level. CellProfiler outputs produced for each cell were then processed using a custom KNIME-based workflow, normalizing and grouping the obtained measurements.

## Data Availability

The raw data is now made available under an open-source license (61).
